# In-stem molecular beacon targeted to a 5′-region of tRNA inclusive of the D arm that detects mature tRNA with high sensitivity

**DOI:** 10.1371/journal.pone.0211505

**Published:** 2019-01-29

**Authors:** Yuichi Miyoshi, Takashi Ohtsuki, Hiromu Kashida, Hiroyuki Asanuma, Kazunori Watanabe

**Affiliations:** 1 Graduate School of Natural Science and Technology, Okayama University, Okayama, Japan; 2 Graduate School of Interdisciplinary Science and Engineering in Health Systems, Okayama University, Okayama, Japan; 3 Graduate School of Engineering, Nagoya University, Nagoya, Japan; University of Toronto, CANADA

## Abstract

Cellular functions are regulated by the up- and down-regulation and localization of RNA molecules. Therefore, many RNA detection methods have been developed to analyze RNA levels and localization. Molecular beacon (MB) is one of the major methods for quantitative RNA detection and analysis of RNA localization. Most oligonucleotide-based probes, including MB, are designed to target a long flexible region on the target RNA molecule, e.g., a single-stranded region. Recently, analyses of tRNA localization and levels became important, as it has been shown that environmental stresses and chemical reagents induce nuclear accumulation of tRNA and tRNA degradation in mammalian cells. However, tRNA is highly structured and does not harbor any long flexible regions. Hence, only a few methods are currently available for detecting tRNA. In the present study, we attempted to detect elongator tRNA^Met^ (eMet) and initiator tRNA^Met^ (iMet) by using an in-stem molecular beacon (ISMB), characterized by more effective quenching and significantly higher sensitivity than those of conventional MB. We found that ISMB1 targeted a 5′- region that includes the D arm of tRNA and that it detected eMet and iMet transcripts as well as mature eMet with high sensitivity. Moreover, the analysis revealed that the formation of the ISMB/tRNA transcript complex required more time than the formation of an ISMB/unstructured short RNA complex. These results suggest that ISMB-based tRNA detection can be a useful tool for various biological and medical studies.

## Introduction

Up- and down-regulation of expression and localization of RNA molecules are associated with the regulation of cellular functions [1,2]. For instance, the up- and down-regulation of mRNA and miRNA levels regulates cell differentiation [3]. The localization of mRNA contributes to the regulation of local translation at the synapse [4,5]. Therefore, RNA detection methods, such as northern blotting, reverse-transcription polymerase chain reaction, microarray analysis, fluorescent probes, and fluorescence *in situ* hybridization (FISH), have been developed to analyze RNA levels and localization [6–9].

Methods using fluorescent probes, such as molecular beacons (MBs), have been developed as one of the major RNA detection approaches [10]. MB, which was first reported by Tyagi et al. [11], is a hairpin oligonucleotide with a fluorophore and a quencher in close proximity. Fluorescence of the fluorophore is quenched in the absence of target RNA. In the presence of target RNA, MB hybridizes with it by opening a stem region, and thereby the fluorescence intensity increases. MB can be used for quantitative RNA detection both *in vitro* and *in vivo*, and for evaluation of RNA localization [9,12,13].

It has been recently shown that initiator tRNA^Met^ (iMet) is degraded by exonucleases Xrn1 and Xrn2, to suppress translation in heat-stressed HeLa cells [14,15]. In addition, it has been shown that iMet and elongator tRNA^Met^ (eMet) accumulate in the nucleus in heat-stressed HeLa cells, and then localize in the nuclear stress bodies [16]. Further, tRNAs accumulate in the nucleus of puromycin-treated Chinese hamster ovary cells [17]. In these studies, northern blotting, FISH, and fluorescently labeled tRNA were mainly employed for quantitative tRNA detection and observation of tRNA localization, since only a few methods are currently available for the detection of tRNA molecules in mammalian cells.

**Fig 1:**
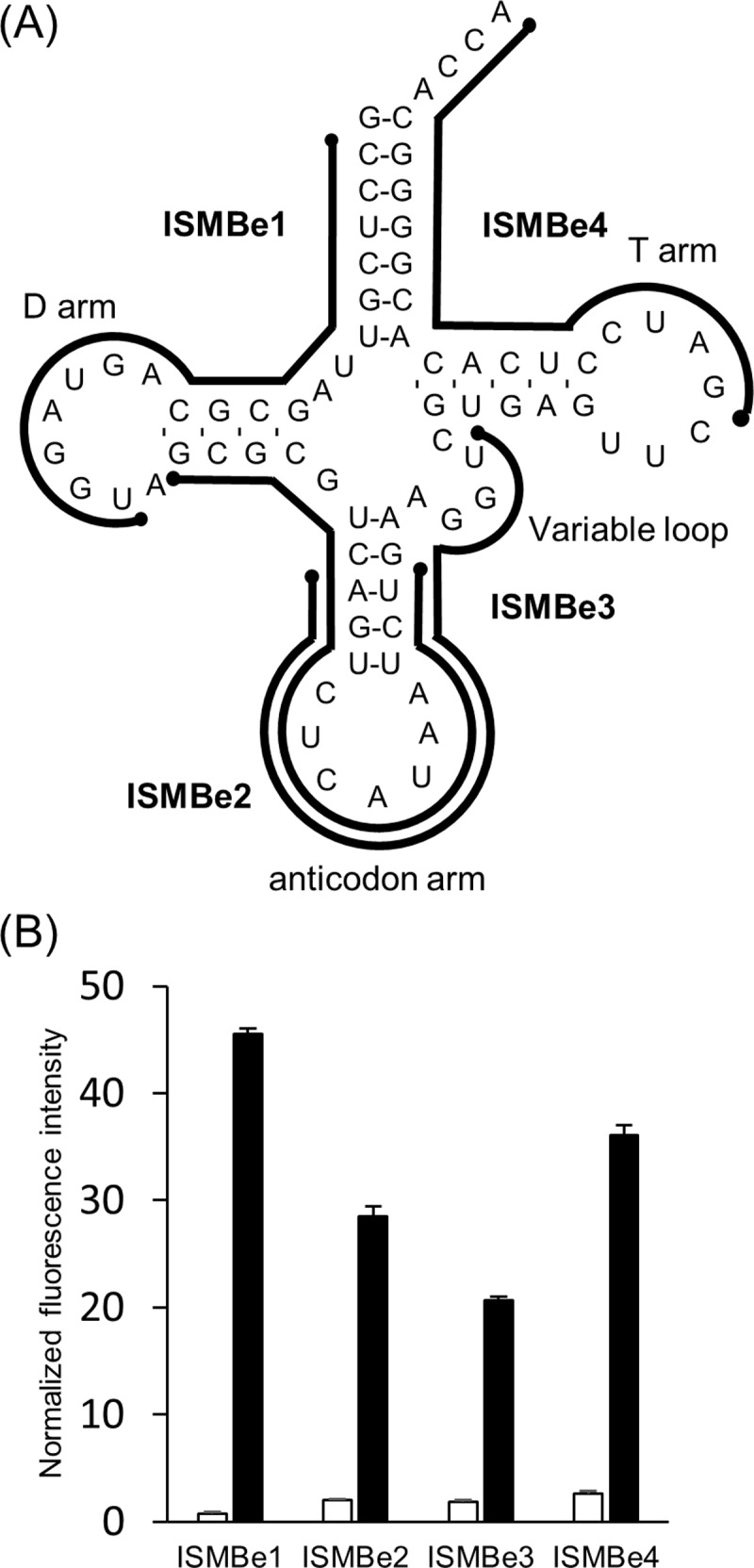
Detection of the eMet transcript by ISMBe. (A) The cloverleaf structure of eMet transcript is shown. Black lines are target regions of the designed ISMBe molecules. (B) The fluorescence intensities of ISMBe probes in the absence of target RNA (white bar) or in the presence of eMet transcript (black bar). The fluorescence intensities were normalized to digested ISMBe1. Data are the means ± standard error of the mean (SEM) of three independent experiments.

Most oligonucleotide-based probes, including MB, are designed to target a long flexible region on the target RNA molecule, such as a single-stranded region [9]. On the other hand, tRNA is highly structured and does not harbor any long flexible regions (Fig 1A). Therefore, detecting tRNA molecules by using oligonucleotide-based probes, such as MB, might be difficult. To overcome this problem, we have recently attempted to detect tRNA transcripts by using conventional MBs targeting the various regions of a tRNA molecule [18]. We have previously shown that conventional MB that targets the D arm of a tRNA^Lys3^ transcript can detect the tRNA^Lys3^ transcript with higher sensitively than MBs targeted to the anticodon arm and the T arm. However, detection sensitivity of a tRNA transcript by using conventional MB was low. Furthermore, it remains unknown whether MB can detect mature tRNA molecules that contain multiple modified nucleotides because modified nucleotides might affect the hybridization with MB. Accordingly, we proposed to employ an in-stem molecular beacon (ISMB) that targets another region of tRNA (Fig 1A). ISMB, in which two fluorophores and four quenchers are incorporated into the stem region as pseudo-base pairs, exhibit effective quenching and significantly higher sensitivity than conventional MB bearing a single fluorophore at one end and a single quencher at the other end of the stem region (S1 Fig) [19–22]. The strong quenching of ISMB is considered to be due to the pseudo-base pairing of fluorophore(s) and quencher(s). In the current study, we designed eight ISMB molecules for detecting iMet, eMet transcript, and mature eMet. We identified the best target region that enabled ISMB to detect tRNA with high sensitivity and specificity. In addition, we demonstrated that the formation of the ISMB/tRNA transcript complex requires more time than ISMB complex formation with unstructured short RNA.

## Materials and methods

### Synthesis of ISMB molecules

Phosphoramidite monomers bearing fluorophore (pentamethyl indocarbocyanine, Cy3) and quencher (nitro methyl red) were synthesized as described previously [22,23]. These monomers were directly used for the synthesis of ISMB via DNA/RNA synthesizer. ISMB molecules, consisting of DNA modified with Cy3 and nitro methyl red, were purchased from Tsukuba oligo service (Ibaraki, Japan). ISMBe and ISMBi target eMet and iMet, respectively. Sequences of ISMB molecules are shown in S1 Table.

### Preparation of eMet, iMet, and mutated eMet transcripts

For the study, eMet, iMet, and mutated eMet transcripts were prepared by *in vitro* transcription. To generate DNA templates for the transcription, primer extension was performed using 2 μM of each primers (shown in S2 Table) in a 100-μl reaction mixture containing 0.2 mM dNTPs and 25 U KOD Dash DNA polymerase (Toyobo, Osaka, Japan), with the following temperature program: 94°C for 30 s; followed by 5 cycles of 94°C for 30 s, 55°C for 5 s, and 74°C for 30 s. The resultant template DNA was precipitated with 2-propanol. T7 RNA polymerase was produced in *Escherichia coli* BL21 pLysS (TaKaRa, Shiga, Japan) and purified on an Ni-NTA column (QIAGEN, Hilden, Germany). The Ni-NTA column was equilibrated with buffer A containing 50 mM Hepes-KOH (pH 7.6), 1 M NH_4_Cl, 10 mM M_g_Cl_2_, and 7 mM 2-mercaptethanol. T7 RNA polymerase was eluted by using a 20–500 mM imidazole gradient, and protein fractions were collected and analyzed by sodium dodecyl sulfate-polyacrylamide gel electrophoresis (SDS-PAGE). Fractions containing T7 RNA polymerase were dialyzed in buffer B containing 50 mM Hepes-KOH (pH 7.6), 100 mM KCl, 10 mM MgCl_2_, 7 mM 2-mercaptethanol, and 30% (v/v) glycerol. The transcription reaction was performed at 37°C for 5 h in a reaction mixture containing 40 mM Tris-HCl (pH 8.0), 24 mM MgCl_2_, 5 mM dithiothreitol, 10 mM GMP, 2 mM each of NTP, 1.8 U/ml inorganic pyrophosphatase (Sigma, St. Louis, MO, USA), 26.2 μg/ml purified T7 RNA polymerase, and 10 μg/ml DNA template. Finally, tRNA transcripts were purified by resolving on an 8% denaturing PAGE.

### Detection of eMet, iMet, and mutated eMet transcripts by ISMB

For the experiment, eMet or iMet transcripts (2 μM) were renatured by incubating for 1 min at 85°C in buffer C containing 10 mM Tris-HCl (pH 8.0) and 2.5 mM MgCl_2_, followed by slow cooling to 4°C. Renatured tRNA transcript (2 μM) was incubated with 100 nM ISMBe or ISMBi in buffer C at 37°C for 2 h. Mutated eMet transcript (500 nM) was renatured and then incubated with 50 nM ISMBe. The fluorescence spectra of ISMB molecules were acquired at an excitation wavelength of 540 nm and an emission wavelength in the range of 550–700 nm using an FP-6600 spectrophotometer (JASCO, Tokyo, Japan). Excitation wavelength was set to ±5 nm bandwidth. To normalize the fluorescence intensities, completely digested 100 nM ISMBe1 was prepared as follows: ISMBe1 was incubated at 37°C for 30 min in a reaction mixture containing 10 mM Tris-HCl (pH 8.0), 2.5 mM MgCl_2_, and 0.15 U/ml DNase I (Roche, Basel, Switzerland). Data are presented as the means ± SEM of three independent experiments.

### Time-dependent detection of eMet transcript and short RNA molecules by ISMBe

Renatured eMet transcript (2 μM) or short RNA molecules were incubated with 100 nM ISMBe in buffer C at 37°C for 10–480 min. The fluorescence spectra were acquired as described above. Short RNA-1 (5ʹ-GCC UCG UUA GCG CAG UAG GU-3ʹ), short RNA-2 (5ʹ-GCG CGU CAG UCU CAU AAU CU-3ʹ), and short RNA-4 (5ʹ-GAU CCU CAC ACG GGG CAC CA-3ʹ) were the same as sequences of each ISMBe target region of eMet. These short RNAs were purchased from JBioS (Saitama, Japan). The fluorescence intensities were normalized to digested 100 nM ISMBe1. Data are presented as the means ± SEM of three independent experiments.

#### Detection limit of ISMBe

Renatured eMet transcript (0–1000 nM) was incubated with 50 nM ISMBe in buffer C at 37°C for 2 h. The fluorescence spectra were acquired as described above. The limit of detection was determined by using the equation: detection limit = [3 × standard deviation (average at 0 nM)]/(slope). The detection limit was calculated based on the linear range (0–500 nM eMet transcript), with concentrations of 1000 nM eMet excluded due to non-linearity. Data are presented as the means ± SEM of three independent experiments.

### Detection of the ISMBe1/tRNA transcript complex and endogenous eMet in living HeLa cells

HeLa cells were obtained from RIKEN BRC which is participating the National Bio-Resource Project of the MEXT, Japan. HeLa cells were maintained in RPMI 1640 medium with 10% fetal bovine serum (Sigma) and 1% antibiotic-antimycotic solution (Gibco, Gaithersburg, MD, USA) at 37°C under an atmosphere of 5% CO_2_. HeLa cells were plated on collagen-coated glass-based dish. After 2 d, the medium was exchanged for fresh medium without phenol red, and the cells were then microinjected with the reaction mixture specified below containing 20 μM Cy5 C5 maleimide (Thermo Fisher Scientific, Waltham, MA, USA) as an injection marker. For the experiment, 10 μM eMet or iMet transcripts was renatured by incubating for 1 min at 85°C in buffer D containing 20% Dulbecco’s phosphate buffer saline (-) and 1 mM MgCl_2_, followed by slow cooling to 4°C. Each renatured tRNA transcript (10 μM) was incubated with 1 μM ISMBe1 in buffer D at 37°C for 2 h. Microinjections were performed using Eppendorf Femtojet microinjection equipment (Eppendorf, Hamburg, Germany) and Femtotip microinjection capillary tips at 70 hPa. All imaging experiments were performed using an Olympus FV1200 confocal microscope (Olympus, Tokyo, Japan). The fluorescence intensities of ISMBe1 and Cy5 were quantified by using FV10-ASW 4.2 software (Olympus). Data are presented as the means ± SEM of four independent experiments. *p*-values were calculated using Student’s *t*-test.

To detect endogenous eMet in living cells, the cells were microinjected with scrambled ISMB or ISMBe1. Imaging experiments were performed using an Olympus IX51 inverted microscope. The fluorescence intensities of scrambled ISMB and ISMBe1 were quantified using FV10-ASW 4.2 software. Data are the means ± SEM of 7 cells for scrambled ISMB and 10 cells for ISMBe1.

### Partial purification of mature eMet from HeLa cells

HeLa cells were grown until 70–80% confluence. The cells were suspended in solution D [4 M guanidium thiocyanate, 25 mM sodium citrate (pH 7.0), 0.5% sarcosyl, and 0.1 M 2-mercaptoethanol] for total RNA extraction. Proteins in the total RNA fraction were removed by phenol-saturated sodium acetate (pH 5.2). Total RNA was collected by precipitation with 2-propanol. It was then applied to a Q-Sepharose column (GE Healthcare, Hilden, Germany) equilibrated with buffer E containing 25 mM Tris-HCl (pH 7.4), 150 mM KCl, and 2.5 mM MgCl_2_. Then, tRNA molecules were eluted with a linear gradient of KCl (0.3–2 M) in buffer E, and the fractions were analyzed by 7 M urea 8% PAGE. The eMet-specific radioisotope-labeled probe was prepared by using [γ-^32^P]-ATP (PerkinElmer, Waltham, MA, USA) and T4 polynucleotide kinase (Toyobo). The sequence of the eMet-specific probe was as follows: 5ʹ-ATT TTT GGT GCC CCG TGT GAG GAT CGA AC-3ʹ. The tRNA mixture (2 μg) containing each fraction eluted from Q-Sepharose was hybridized with 8.5 pmol eMet-specific radioisotope-labeled probe in a buffer containing 10 mM Tris-HCl (pH 6.8) and 0.5 mM MgCl_2_ for 10 min at 95°C, followed by slow cooling to 25°C. The reaction mixture was resolved on 8% native PAGE, and then eMet/probe complex was detected by using BIOMAX MS Film (Sigma). The fraction containing mature eMet was collected and the concentration of mature eMet was calculated using ImageJ software (Rasband, W.S., ImageJ, U. S. National Institutes of Health, Bethesda, MD, USA, https://imagej.nih.gov/ij/, 1997–2018), by comparison with the band intensity of 8.5 pmol eMet transcript/probe complex.

### Detection of mature eMet by ISMBe

Renatured mature eMet (500 nM) was incubated with 50 nM ISMBe in buffer C at 37°C for 2 h. The fluorescence spectra were acquired as described above. Data are presented as the means ± SEM of three independent experiments.

## Results

### Detection of eMet and iMet transcripts by ISMB

Secondary structure of the eMet transcript was shown in Fig 1A. To detect the eMet transcript, we designed four ISMBe molecules (Fig 1A). ISMBe1 was targeted to the 5ʹ-region, including the D arm; ISMBe2 was targeted to the anticodon arm, excluding the variable loop; ISMBe3 was targeted to the anticodon arm, including the variable loop; and ISMBe4 was targeted to the 3ʹ-region including the T arm. To confirm that each ISMBe molecule detected the eMet transcript, the fluorescence intensities of ISMBe molecules were determined in the presence of eMet transcript. As shown in Fig 1B, each ISMBe molecule could detect eMet transcript, with ISMBe1 showing the greatest fluorescence increase. In the absence of eMet transcript, the fluorescence intensities of ISMBe molecules remained at the background level. To investigate whether this behavior would also be observed with other tRNA molecules, we designed ISMB molecules to target iMet (ISMBi). The numerals of ISMBi correspond to the target region of ISMBe (S2A Fig). As shown in S2B Fig, ISMBi1 exhibited the highest sensitivity. These observations indicated that the target region including the D arm was the most suitable region for detecting a tRNA transcript by ISMB. On the other hand, the target region including the variable loop, such as ISMB3, was not suitable. Therefore, ISMBe molecules other than ISMBe3 were used in the subsequent analyses.

### Time-dependent increase of fluorescence intensity in the presence of eMet transcript or unstructured short RNA molecule

**Fig 2:**
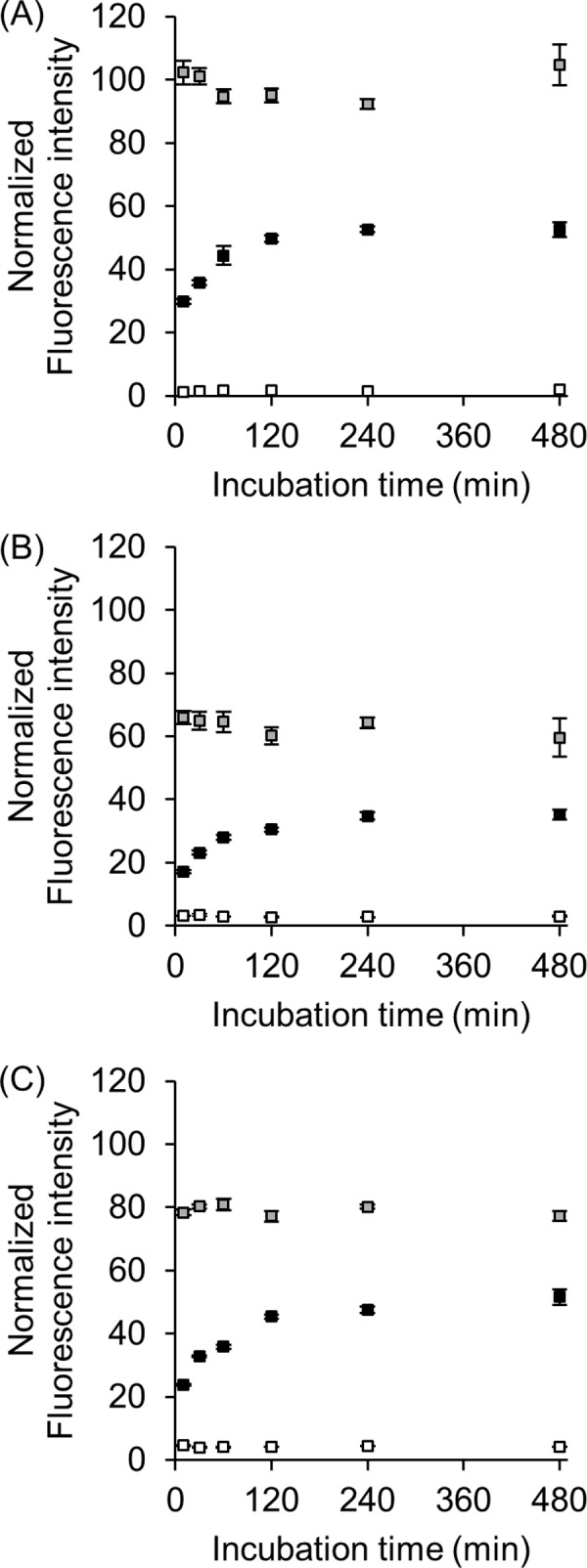
Time-dependent increase of the fluorescence intensity of ISMBe in the presence of the target RNA molecule. ISMBe1 (A), ISMBe2 (B), and ISMBe4 (C) were used in the experiments. The fluorescence intensities of ISMBe in the absence of target RNA (white), in the presence of short RNA (gray), and eMet transcript (black) are shown. The fluorescence intensities were normalized to digested ISMBe1. Data are the means ± SEM of three independent experiments.

We previously showed that the fluorescence intensity of conventional MB in the presence of tRNA^Lys3^ transcript (15-min reaction) was lower than that of ISMBe and ISMBi in the presence of tRNA transcript (2-h reaction) (Figs 1B and S2B) [18]. These observations suggested that the formation of the ISMBe/tRNA transcript complex requires a relatively long time. We reasoned that the binding of MB to tRNA molecules in a short period time could be difficult, as the latter are highly structured and typically do not have single-stranded regions. To test this working hypothesis, time-dependent fluorescence intensities of ISMBe were measured in the presence of the target RNA. The fluorescence intensities of ISMBe in the absence of the target RNA remained at the background level, with ISMBe quenched for 480 min (Fig 2A–2C). In the presence of eMet transcript, the fluorescence intensities of ISMBe increased in a time-dependent manner and reached a plateau in 120 min (Fig 2A–2C). By contrast, the fluorescence intensities of ISMBe reached a plateau already after 10 min in the presence of short RNA molecules (Short RNA-1, -2, -4), whose sequences were exactly the same as those of the target regions of eMet (Fig 2A–2C). These observations suggested that ISMBe could rapidly hybridize with short RNA, as compared with the eMet transcript. As a consequence, the fluorescence increase in the presence of unstructured RNA was faster than that in the presence of tRNA transcript. In other words, these observations indicated that the formation of the ISMB/RNA complex depends on the rigidity of RNA structure.

### The detection limit of ISMBe1 and ISMBe4 is lower than that of ISMBe2

**Fig 3:**
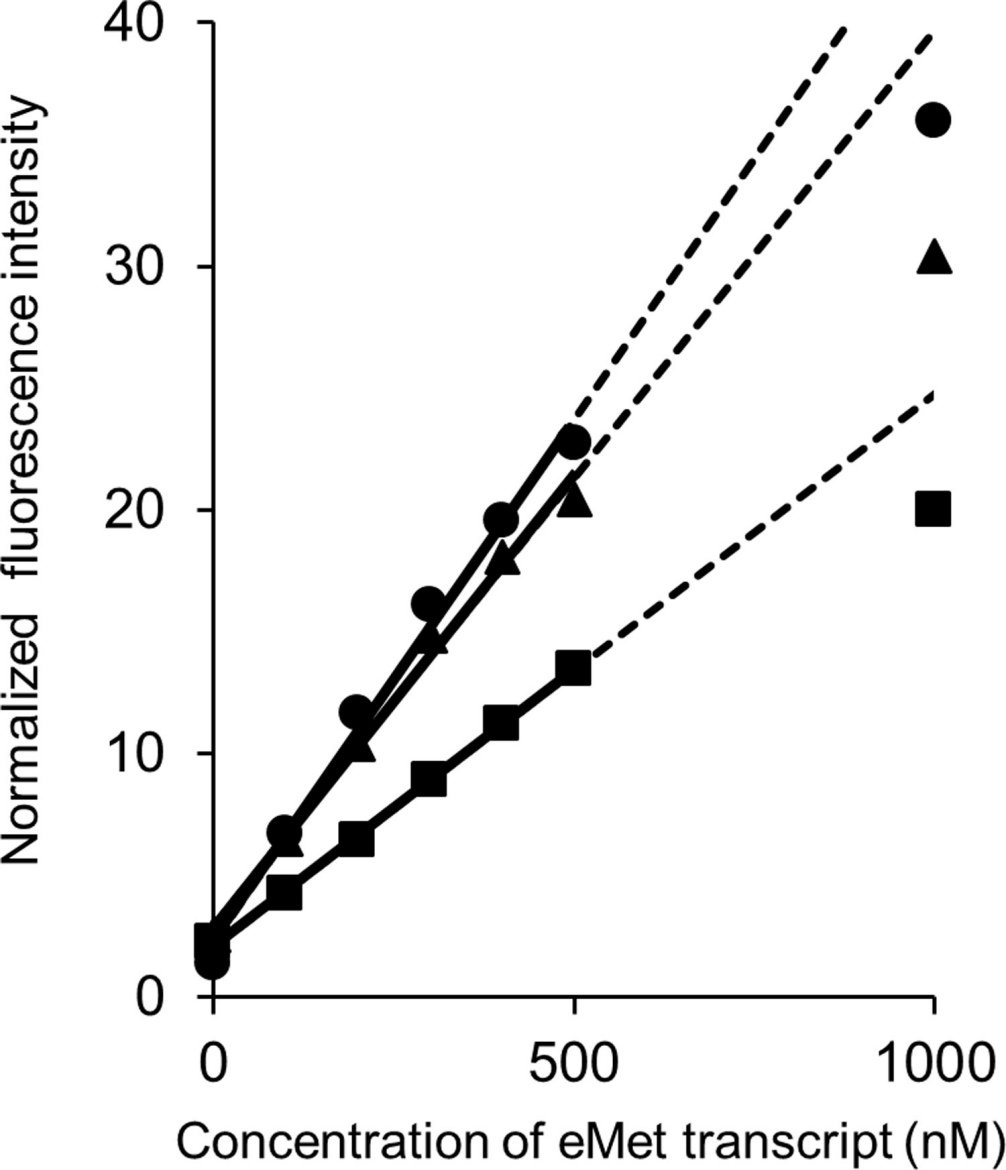
Detection limit of ISMBe molecules. ISMBe1 (circle), ISMBe4 (triangle), and ISMBe2 (square) were used in the experiments. Linear trendlines were plotted using the data points of fluorescence at 0–500 nM concentrations of the eMet transcript.

To determine the detection limit of ISMBe, the linearity of fluorescent response of each ISMBe molecule to a target transcript was examined. The fluorescence of each ISMBe molecule responded linearly to the increasing concentrations of the eMet transcript up to 500 nM (Fig 3). The detection limit of ISMBe1 and ISMBe4 was 26.6 nM and 22.6 nM, respectively. The detection limit of ISMBe2 was 63.7 nM. These observations indicated that ISMBe1 and ISMBe4 were able to detect the eMet transcript with greater sensitively than ISMBe2.

### ISMBe specifically detects eMet transcript

**Fig 4:**
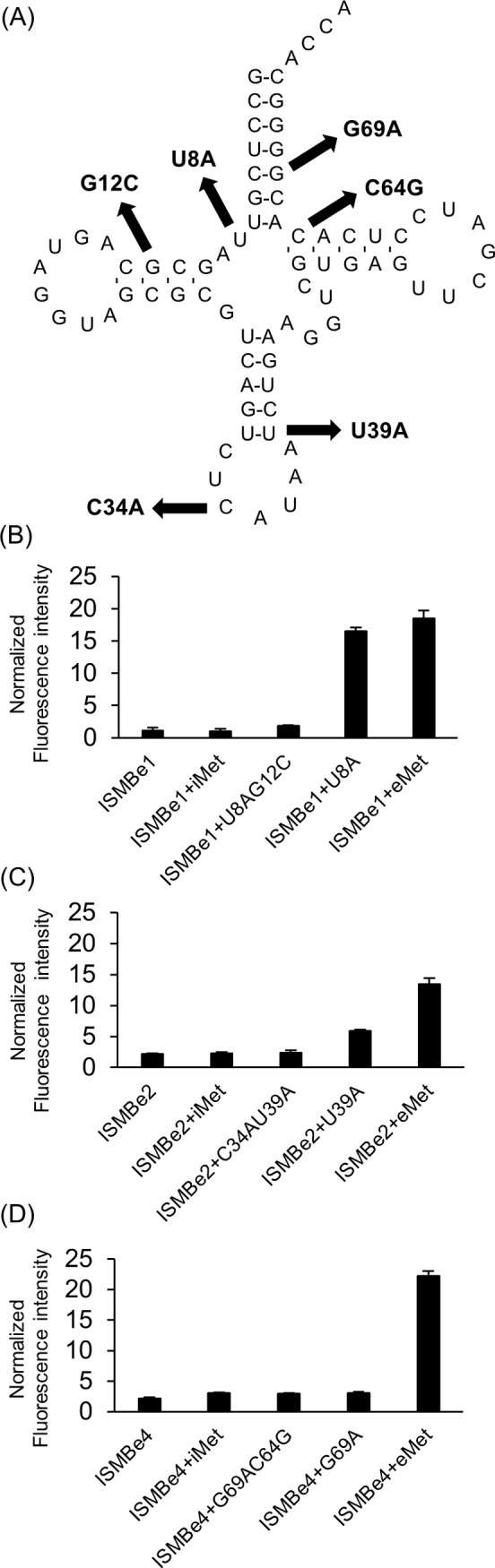
The specificity of ISMBe molecules. (A) Point mutations were individually introduced into the eMet transcript. The arrows indicate the mutation site and the substituted nucleotides. The fluorescence intensities of ISMBe1 (B), ISMBe2 (C), and ISMBe4 (D) in the presence of eMet transcript and mutated eMet transcripts are shown. Data are the means ± SEM of three independent experiments.

To confirm that ISMBe specifically hybridized with the eMet transcript, eMet transcripts with one- and two-base mismatches were prepared (Fig 4A). In addition, iMet transcript, which is accumulated in the nucleus after transfer from the cytoplasm, was also prepared as its sequence differs from that of eMet [16]. As shown in Fig 4B–4D, the fluorescence intensities of each ISMBe molecule in the presence of the iMet transcript and eMet transcripts with two-base mismatches (U8AG12C, C34AU39A, and G69AC64G) remained at the background level. Moreover, the fluorescence intensity of ISMBe4 increased only in the presence of the eMet transcript (Fig 4D). On the other hand, the fluorescence intensity of ISMBe2 slightly increased in the presence of mutated transcript U39A, and the fluorescence intensity of ISMBe1 in the presence of mutated transcript U8A was similar to that in the presence of eMet transcript (Fig 4B and 4C). These observations indicated that ISMBe4 was highly specific to eMet transcript.

### Detection of the ISMBe1/eMet transcript complex in living HeLa cells

**Fig 5:**
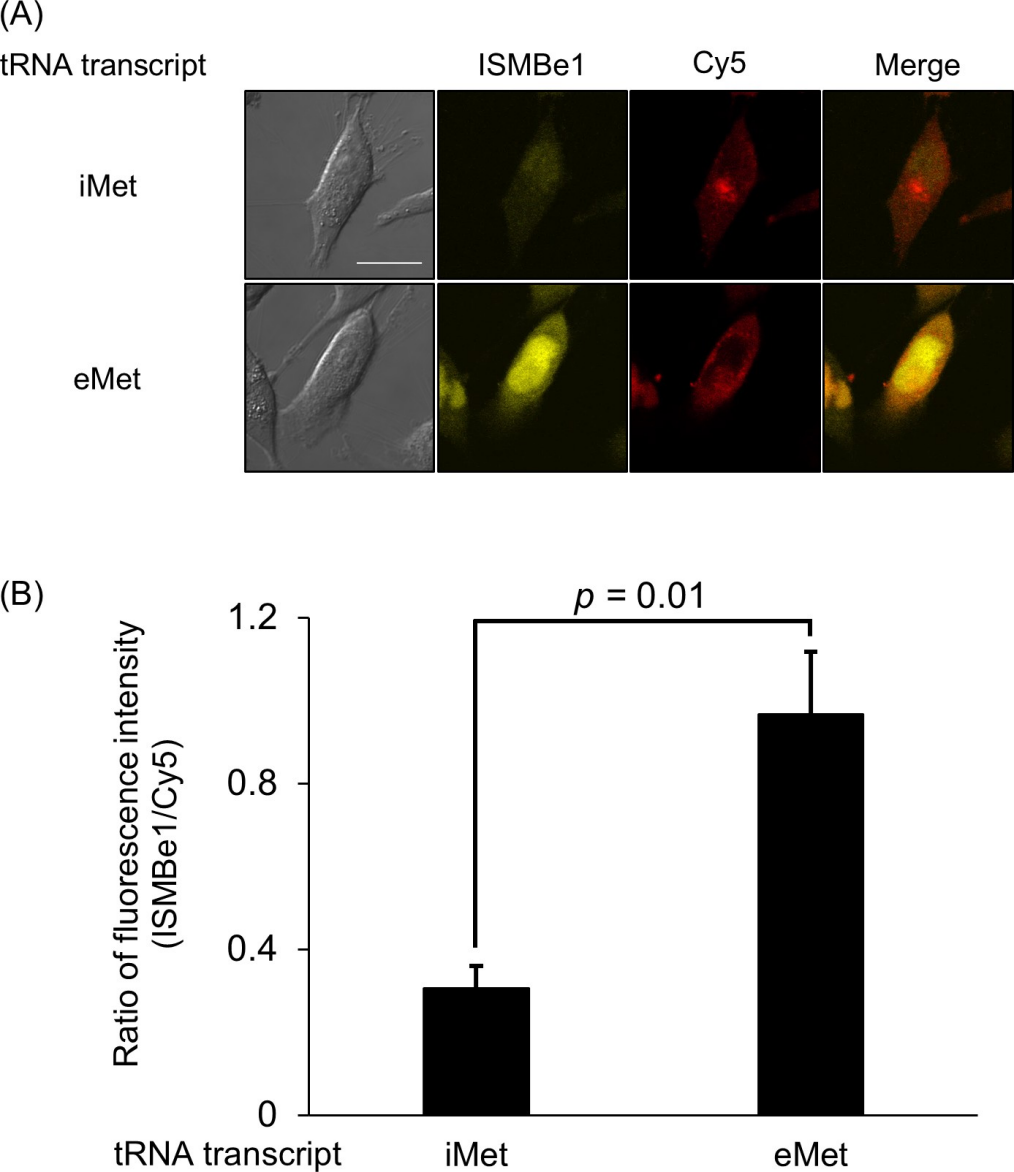
Detection of the ISMBe1/eMet transcript complex in living cells. (A) Detection of the ISMBe1/eMet transcript complex in living cells, with Cy5 introduced as an injection marker. (B) The fluorescence intensity ratio of ISMBe1 in the presence of iMet transcript (left) and in the presence of eMet transcript (right). The fluorescence intensities were normalized to Cy5. Scale bar, 20 μm. Data are the means ± SEM of four independent experiments. More than 6 cells were measured in each experiment. *p*-values were calculated using Student’s *t*-test.

As shown in Fig 1B, the signal background ratio of ISMBe1 was the highest. Therefore, ISMBe1 was initially used to detect endogenous eMet in living cells. To confirm the formation of a complex between ISMBe1 and the eMet transcript in living cells, ISMBe1 and eMet transcript were introduced into HeLa cells by microinjection. For the experiment, the iMet transcript was injected with ISMBe1 as a negative control. As shown in Fig 5A and 5B, the fluorescence intensity of ISMBe1 in the cells after eMet transcript injection was significantly higher than that in cells after iMet transcript injection. This observation suggested that ISMBe1 is able to detect mature eMet in living HeLa cells.

To detect endogenous eMet in living cells, ISMBe1 and scrambled ISMB were introduced into HeLa cells by microinjection. The fluorescence intensities of ISMBe1 and scrambled ISMB in living cells were similar at 120 min (S3A Fig). Next, time-dependent fluorescence intensities of ISMBe1 and scrambled ISMB were measured in living cells (S3B Fig). The fluorescence intensities of both molecules increased to a similar extent in a time-dependent manner at similar level in living cells. However, the fluorescence intensity of ISMBe1 in the absence of the target RNA remained at background levels until 480 min (Fig 2A), indicating that ISMB may be degraded in living cells.

### ISMBe detects mature eMet

**Fig 6:**
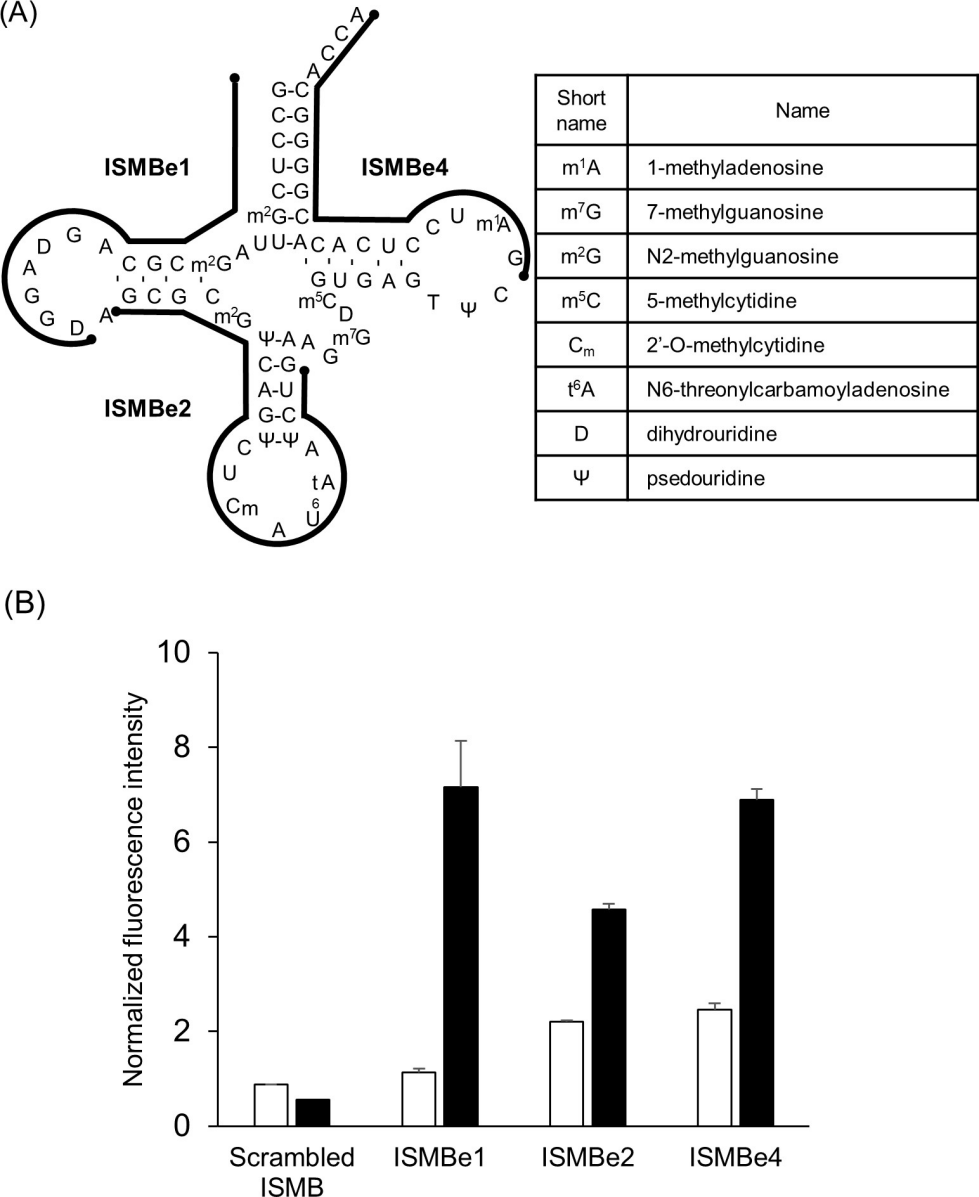
Detection of mature eMet by ISMBe. (A) The cloverleaf structure of mature eMet is shown [25]. (B) The fluorescence intensities of ISMBe molecules in the absence of target RNA (white) and in the presence of mature eMet (black). The fluorescence intensities were normalized to digested ISMBe1. Data are the means ± SEM of three independent experiments.

We were concerned that multiple nucleotide modifications of mature eMet might interfere with the hybridization of ISMBe1 and mature eMet (Fig 6A), since the presence of modified nucleotides in tRNA interferes with the hybridization of a DNA array probe and tRNA [24]. To address this question, we initially prepared partially purified mature eMet from HeLa cells. Total RNA from HeLa cells was fractionated by anion-exchange chromatography and tRNA molecules in each fraction were detected by electrophoresis. We observed that tRNA molecules were present in fractions 13–16 (S4A Fig). To identify the fraction that contained mature eMet, eMet-specific radioisotope-labeled probe was used. The eMet/probe complex was detected only in fraction 13, indicating that mature eMet was contained in that fraction (S4B Fig).

To investigate whether ISMBe could detect mature eMet, the fluorescence intensities of ISMBe molecules were determined in the presence of mature eMet from fraction 13. The fluorescence intensity of scrambled ISMB in the presence of mature eMet remained at the background level (Fig 6B). The fluorescence intensities of ISMBe molecules in the presence of mature eMet increased, with ISMBe1 exhibiting the highest fluorescence increase. However, the increase of fluorescence of ISMBe molecules in the presence of mature eMet was lower than that of ISMBe molecules in the presence of eMet transcript (Figs 1B and 6B). These observations indicated that ISMBe molecules can be used to detect mature eMet, however, modified nucleotides impact the hybridization of ISMBe molecules and mature eMet.

## Discussion

In the current study, to determine the effective target region of ISMB for detecting tRNA molecules, eight ISMB molecules targeted to the representative tRNA regions were designed. We showed that ISMBe molecules, particularly ISMBe1, targeted to the 5ʹ-region inclusive of the D arm, could be used to detect eMet transcript and mature eMet (Figs 1 and 6). In addition, ISMBi1, targeted to the 5ʹ-region inclusive of the D arm, similarly detected the iMet transcript with higher sensitivity than the other ISMBi probes (S2B Fig). These observations indicated that ISMB targeted to the 5ʹ-region, inclusive of the D arm, detected tRNA with high sensitivity.

To determine whether the sensitivity of ISMB molecules depends on the stability of the ISMB/tRNA complex, we calculated the free energy (ΔG) of each ISMBe and ISMBe/eMet transcript complex using the RNAstructure 5.3 software (S3 Table) [26,27]. The predicted free energies were the same, indicating lack of correlation between the sensitivity of ISMB and the stability of the ISMBe/eMet transcript complex. In a previous study, we showed that conventional MB molecules targeted to the D arm more sensitively detect tRNA^Lys3^ transcript than those targeted to the anticodon arm and the T arm [18]. Furthermore, according to thermal unfolding studies, the D arm is more readily melted than other regions [28,29]. This indicated that ISMB most readily hybridizes with the D arm.

Previously, we showed that the fluorescence increase of conventional MB targeted to the T arm, excluding the CCA sequence, is the lowest among the analyzed probes targeting various tRNA regions [18]. On the other hand, the fluorescence increases of ISMBe4 and ISMBi4 molecules targeted to the T arm, including the CCA sequence, in the presence of eMet and iMet transcript, respectively, were higher than those of ISMBe2 and ISMBi2 targeted to the anticodon arm, respectively (Figs 1B and S2B). Furthermore, the detection limit of ISMBe1 and ISMBe4 was almost the same (Fig 3). Although the tRNA molecule is highly structured, the CCA sequence of tRNA is unfolded and forms a short single-stranded region. As shown in Fig 2, ISMBe could rapidly hybridize with short RNA. Collectively, these observations suggested that ISMBe4 initially hybridizes with the CCA sequence of tRNA and then hybridizes with the target sequence of the T arm region of tRNA.

As shown in Figs 1, 2 and 6, the formation of the ISMB/RNA complex depends on the rigidity of RNA structure, and the modified nucleotides of mature eMet impact the hybridization of ISMBe and mature eMet. Data shown in Figs 1 and 6 indicate an additional possibility. In general, the modified nucleotides on tRNA contribute to the thermal stability of tRNA [30]. For instance, mature tRNA^Asp^ (Tm = 73°C) from yeast is more stable than the tRNA^Asp^ transcript (Tm = 65°C) [31]. Therefore, the hybridization of ISMBe and mature eMet might be inhibited relative to that of the ISMBe and eMet transcript, since eMet transcript melts more easily than mature eMet.

The current study highlights some limitations of the devised method. First, ISMBe1 and ISMBe2 detected eMet transcripts with one-base mismatches (Fig 4B and 4C). In addition, multiple modified nucleotides on a mature tRNA molecule affected the hybridization of ISMB and mature tRNA (Fig 6). Moreover, we found that ISMB may be degraded in living cells (S3 Fig). These issues might be addressed by designing ISMB based on other structural backbones, such as locked nucleic acids (LNAs), peptide nucleic acids (PNAs) and serinol nucleic acids (SNAs) which introduces 2,6-diaminopurine [32–37]. LNA-, PNA-, and SNA-based probes can bind to double-stranded RNA with high affinity and high specificity. Furthermore, these probes are resistant to most enzymes, such as DNase and RNase. Therefore, LNA-, PNA-, or SNA- based ISMB probes may be able to detect endogenous tRNA in living cells without being degraded.

In summary, we demonstrated that ISMB targeted to the D arm region is the most suitable probe for detecting tRNA transcripts and mature tRNA. While the ISMB/short RNA complex was rapidly formed, the formation of the tRNA/ISMB complex required a relatively long time. Until now, tRNA localization has been mainly analyzed by the FISH method and fluorescence labeling of tRNA in mammalian cells [14,16,17,38–40], and tRNA levels are mainly analyzed by northern blotting [14,41]. Therefore, endogenous tRNA molecules have been not yet observed in living mammalian cells. In the future, ISMB, after the above-mentioned issues have been resolved, may be used to detect endogenous tRNA in living mammalian cells. ISMB-based tRNA detection and tRNA localization analysis in mammalian cells exposed to environmental stresses and chemical reagents may constitute a useful tool for various biological and medical applications.

## Supporting information

S1 Fig Structures of ISMB and conventional MB. (TIF)

S2 Fig Detection of the iMet transcript by ISMBi. (A) The cloverleaf structure of iMet transcript is shown. Black lines are target regions of each designed ISMBi molecule. (B) The fluorescence intensities of ISMBi in the absence of target RNA (white bar) or in the presence of eMet transcript (black bar). The fluorescence intensities were normalized to digested ISMBe1. Data are the means ± SEM of three independent experiments.
(TIF)

S3 Fig Detection of endogenous eMet in living cells. (A) Image of living cells injected ISMB at 120 min. (B) Increase in the fluorescence of scrambled ISMB (square) and ISMBe1 (triangle) in living cells are shown. Fluorescence intensity of each cell at 0 min was defined as 1.0. Data are the means ± SEM of 7 cells for scrambled ISMB and 10 cells for ISMBe1. Scale bar, 20 μm.
(TIF)

S4 Fig Partial purification of mature eMet from HeLa cells. (A) After anion exchange chromatography, each fraction was analyzed by 7 M urea 8% PAGE. (B) The complex of eMet with eMet-specific radioisotope-labeled probe was electrophoresed on 8% native PAGE, and was then detected by ethidium bromide staining (left) and by autoradiography (right).
(TIF)

S1 Table ISMB sequences. F, fluorophore; Q, quencher. The underlined bases were designed to hybridize to the target region.
(PDF)

S2 Table Primers used for preparing eMet, iMet, and mutated eMet transcripts. (PDF)

S3 Table ΔG values of each ISMBe and ISMBe/eMet transcript complex. (PDF)
