# Supplementary figures and images for: In-stem molecular beacon targeted to a 5′-region of tRNA inclusive of the D arm that detects mature tRNA with high sensitivity
Source: PLoS One. 2019 Jan 29;14(1):e0211505. doi: 10.1371/journal.pone.0211505 (PMC6351059; doi:10.1371/journal.pone.0211505)

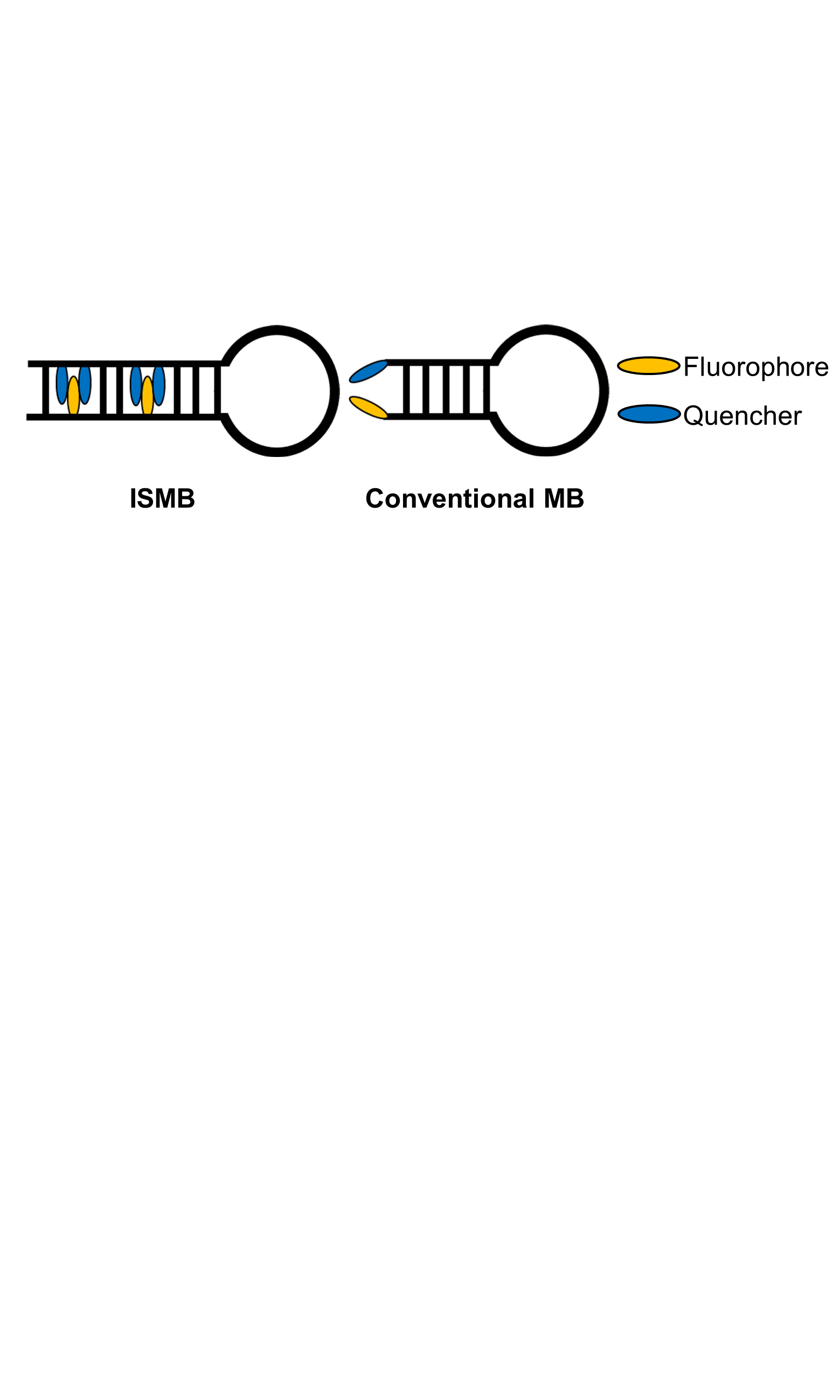

Supplement: S1 Fig — (TIF) [file pone.0211505.s001.tif]

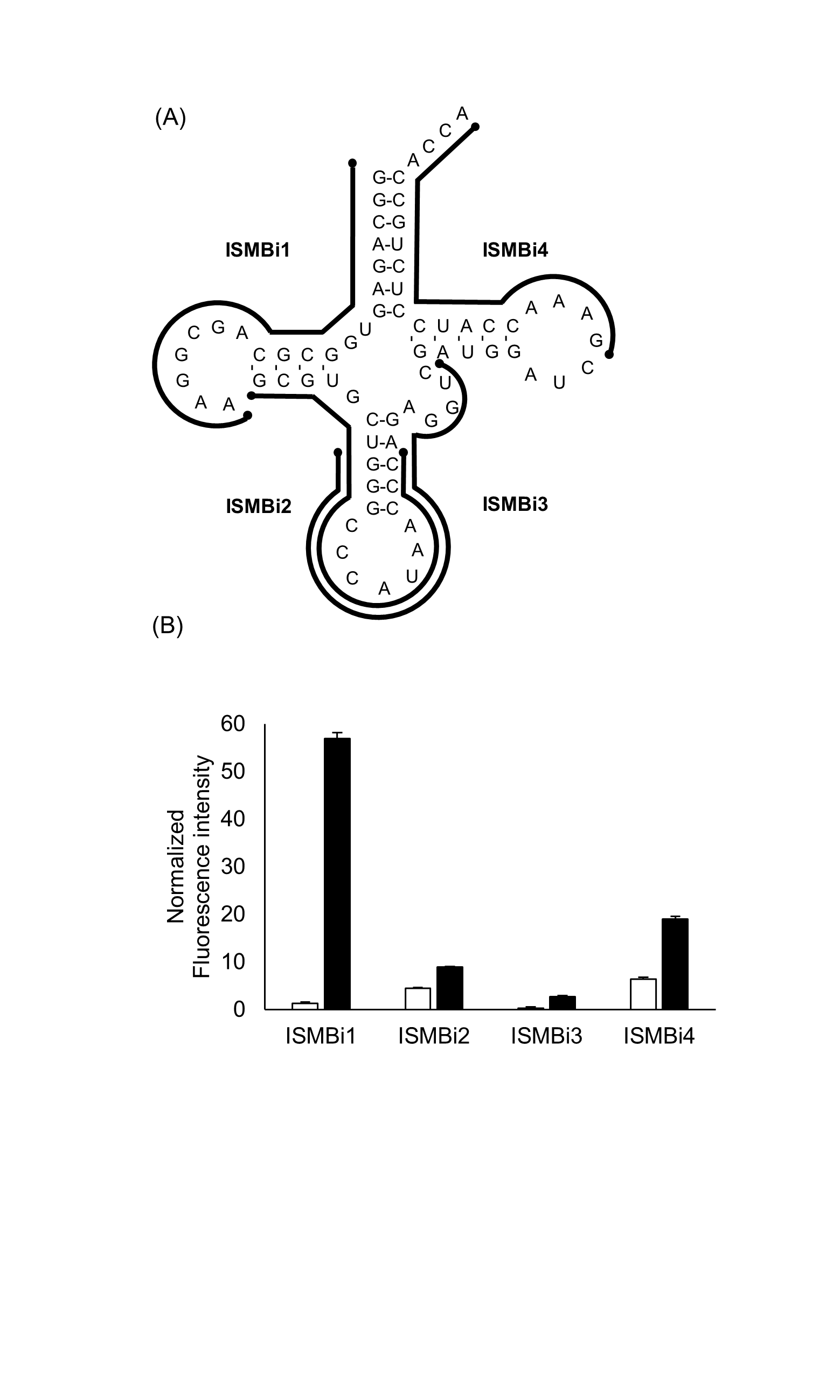

Supplement: S2 Fig — (A) The cloverleaf structure of iMet transcript is shown. Black lines are target regions of each designed ISMBi molecule. (B) The fluorescence intensities of ISMBi in the absence of target RNA (white bar) or in the presence of eMet transcript (black bar). The fluorescence intensities were normalized to digested ISMBe1. Data are the means ± SEM of three independent experiments. (TIF) [file pone.0211505.s002.tif]

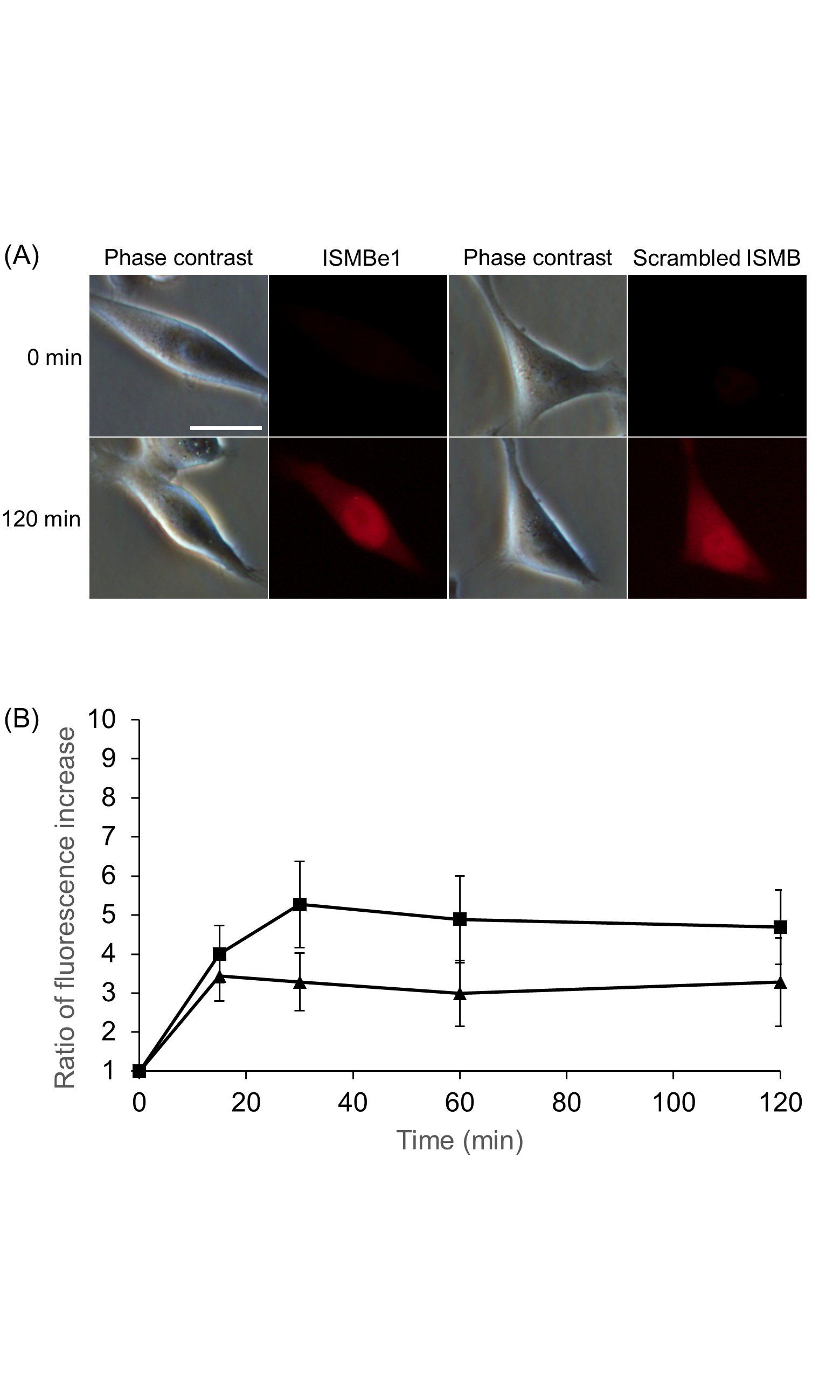

Supplement: S3 Fig — (A) Image of living cells injected ISMB at 120 min. (B) Increase in the fluorescence of scrambled ISMB (square) and ISMBe1 (triangle) in living cells are shown. Fluorescence intensity of each cell at 0 min was defined as 1.0. Data are the means ± SEM of 7 cells for scrambled ISMB and 10 cells for ISMBe1. Scale bar, 20 μm. (TIF) [file pone.0211505.s003.tif]

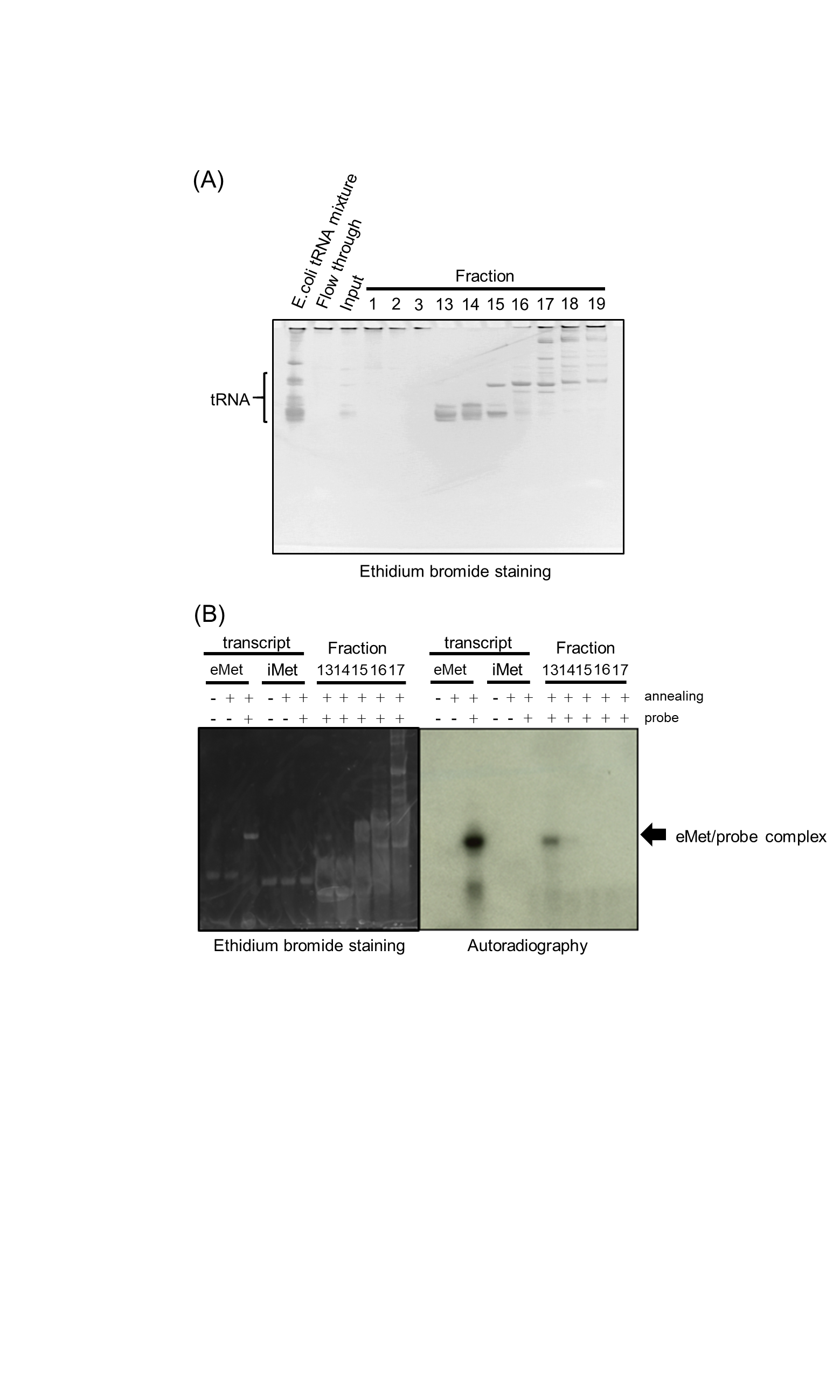

Supplement: S4 Fig — (A) After anion exchange chromatography, each fraction was analyzed by 7 M urea 8% PAGE. (B) The complex of eMet with eMet-specific radioisotope-labeled probe was electrophoresed on 8% native PAGE, and was then detected by ethidium bromide staining (left) and by autoradiography (right). (TIF) [file pone.0211505.s004.tif]
