# Supplementary material for: In-stem molecular beacon targeted to a 5′-region of tRNA inclusive of the D arm that detects mature tRNA with high sensitivity
Source: PLoS One. 2019 Jan 29;14(1):e0211505. doi: 10.1371/journal.pone.0211505 (PMC6351059; doi:10.1371/journal.pone.0211505)
Supplement: S1 Table — F, fluorophore; Q, quencher. The underlined bases were designed to hybridize to the target region. (PDF) [file pone.0211505.s005.pdf]

S1 Table: Sequence of ISMBs

| ISMB | Sequence |
|---|---|
| ISMBe1 | 5'-AFA CCF TAC TGC GCT AAC GAG GCT AQQ GGT QQT-3' |
| ISMBe2 | 5'-AFA GAF TTA TGA GAC TGA CGC GCA AQQ TCT QQT-3' |
| ISMBe3 | 5'-AFG ACF CTT CAG ATT ATG AGA CTA TQQ GTC QQT-3' |
| ISMBe4 | 5'-AFT GGF TGC CCC GTG TGA GGA TCC AQQ CCA QQT-3' |
| ISMBi1 | 5'-AFT TCF CGC TGC GCC ACT CTG CCC GQQ GAA QQT-3' |
| ISMBi2 | 5'-AFG GGF TTA TGG GCC CAG CAC GCA AQQ TCT QQT-3' |
| ISMBi3 | 5'-AFG GAF CCT CTG GGT TAT GGG CCG GQQ TCC QQT-3' |
| ISMBi4 | 5'-AFT GGF TGG CAG AGG ATG GTT TCA CQQCA QQT -3' |
| Scrambled ISMB | 5'-AFG TTF GAT GTG TTT AGT CGC TCT CQQ AAC QQT-3' |
