# Supplementary material for: In-stem molecular beacon targeted to a 5′-region of tRNA inclusive of the D arm that detects mature tRNA with high sensitivity
Source: PLoS One. 2019 Jan 29;14(1):e0211505. doi: 10.1371/journal.pone.0211505 (PMC6351059; doi:10.1371/journal.pone.0211505)
Supplement: S2 Table — (PDF) [file pone.0211505.s006.pdf]

S2 Table. Primer sequences used for preparing eMet, iMet and mutant eMet transcripts

Transcripts	Primers
eMet	(Forward) 5'-CGA AAT TAA TAC GAC TCA CTA TAG CCT CGT TAG CGC AGT AGG TAG CGC GTC AGT CTC ATA ATC-3'<br>(Reverse) 5'-TGG TGC CCC GTG TGA GGA TCG AAC TCA CGA CCT TCA GAT TAT GAG ACT GAC GCG CTA C-3'
U8A	(Forward) 5'-CGA AAT TAA TAC GAC TCA CTA TAG CCT CGT AAG CGC AGT AGG TAG CGC GTC AGT CTC ATA ATC-3'<br>(Reverse) 5'-TGG TGC CCC GTG TGA GGA TCG AAC TCA CGA CCT TCA GAT TAT GAG ACT GAC GCG CTA C-3'
U8AG12C	(Forward) 5'-CGA AAT TAA TAC GAC TCA CTA TAG CCT CGT AAG CCC AGT AGG TAG GGC GTC AGT CTC ATA ATC-3'<br>(Reverse) 5'-TGG TGC CCC GTG TGA GGA TCG AAC TCA CGA CCT TCA GAT TAT GAG ACT GAC GCG CTA C-3'
U39A	(Forward) 5'-CGA AAT TAA TAC GAC TCA CTA TAG CCT CGT TAG CGC AGT AGG TAG CGC GTC AGT CTC ATA AAC-3'<br>(Reverse) 5'-TGG TGC CCC GTG TGA GGA TCG AAC TCA CGA CCT TCA GTT TAT GAG ACT GAC GCG CTA C-3'
C34AU39A	(Forward) 5'-CGA AAT TAA TAC GAC TCA CTA TAG CCT CGT TAG CGC AGT AGG TAG CGC GTC AGT CTA ATA ATC-3'<br>(Reverse) 5'-TGC CCC GTG TGA GGA TCG AAC TCA CGA CCT TCA GAT TAT TAG ACT GAC GCG CTA C-3'
G69A	(Forward) 5'-CGA AAT TAA TAC GAC TCA CTA TAG CCT CGT TAG CGC AGT AGG TAG CGC GTC AGT CTC ATA ATC-3'<br>(Reverse) 5'-TGG TGC CTC GTG TGA GGA TCG AAC TCA CGA CCT TCA GAT TAT GAG ACT GAC GCG CTA C-3'
G69AC64G	(Forward) 5'-CGA AAT TAA TAC GAC TCA CTA TAG CCT CGT TAG CGC AGT AGG TAG CGC GTC AGT CTC ATA ATC-3'<br>(Reverse) 5'-TGG TGC CTC GTG CGA GGA TCG AAC TCG CGA CCTT CAG ATT ATG AGA CTG ACG CGC TAC-3'
iMet	(Forward) 5'-CGA AAT TAA TAC GAC TCA CTA TAG CCT CGT TAG CGC AGT AGG TAG CGC GTC AGT CTC ATA ATC-3'<br>(Reverse) 5'-TGG TGC CCC GTG TGA GGA TCG AAC TCA CGA CCT TCA GAT TAT GAG ACT GAC GCG CTA C-3'
