# Supplementary material for: In-stem molecular beacon targeted to a 5′-region of tRNA inclusive of the D arm that detects mature tRNA with high sensitivity
Source: PLoS One. 2019 Jan 29;14(1):e0211505. doi: 10.1371/journal.pone.0211505 (PMC6351059; doi:10.1371/journal.pone.0211505)
Supplement: S3 Table — (PDF) [file pone.0211505.s007.pdf]

S3 Table. ΔG (kcal mol^{-1}) of each ISMBe and ISMBe/eMet transcript complex

	ΔG of ISMB	ΔG of ISMB/eMet transcript complex
ISMBe1	-7.1	-54.1
ISMBe2	-2.0	-50.0
ISMBe3	-2.6	-52.8
ISMBe4	-8.6	-50.5
ISMBi1	-7.5	-58.4
ISMBi2	-6.0	-57.1
ISMBi3	-10.5	-61.4
ISMBi4	-6.2	-57.8
